# Age and parous-experience dependent changes in emotional contagion for positive infant sounds

**DOI:** 10.3389/fpsyg.2024.1336126

**Published:** 2024-03-27

**Authors:** Nobuaki Mizuguchi, Kenji Kato, Sho K. Sugawara, Tatsuya Yoshimi, Yuta Goto, Kaori Takasu, Tadao Isaka

**Affiliations:** ^1^Research Organization of Science and Technology, Ritsumeikan University, Kusatsu, Shiga, Japan; ^2^Institute of Advanced Research for Sport and Health Science, Ritsumeikan University, Kusatsu, Shiga, Japan; ^3^Assistive Robot Center, National Center for Geriatrics and Gerontology, Obu, Aichi, Japan; ^4^Neural Prosthetics Project, Tokyo Metropolitan Institute of Medical Science, Setagaya, Tokyo, Japan; ^5^Administrative Office, Ritsumeikan University, Kusatsu, Shiga, Japan; ^6^College of Sport and Health Science, Ritsumeikan University, Kusatsu, Shiga, Japan

**Keywords:** well-being, valence, women, emotion, voice, empathy

## Abstract

**Introduction:**

Emotional contagion is achieved by inferring and emotionally resonating with other persons’ feelings. It is unclear whether age-related changes in emotional contagion for infant sounds are modulated by the experience of childbirth or childcare. This study aims to evaluate changes in inference and emotional resonance for positive and negative infant sounds (laughter and crying) among women, based on age and parous experience.

**Methods:**

A total of 241 women (60 young nulliparous, 60 young parous, 60 old nulliparous, and 61 old parous) completed a web-based questionnaire. After listening to three types of infant sounds (laughter, cooing, and crying), participants responded with their valence for hearing infant sounds and estimated infant valence on an 11-point Likert scale.

**Results:**

The analysis for emotional resonance revealed that the correlation coefficient between self and estimated infant valences was greater in young parous and old nulliparous women than in young nulliparous women, in laughter and cooing sounds. However, correlation coefficients for crying did not differ among any of the four groups.

**Conclusion:**

The degree of emotional resonance for infant valence increased depending on age and parous-experience for positive infant sounds.

## 1 Introduction

As humans are a highly social species, we can feel happy when we vicariously share the joy of others, and we can feel suffering when we empathize with someone in pain (Singer and Klimecki, 2014). This phenomenon is called empathy and is associated with successful communication, caring interpersonal relationships, and prosocial behavior (Sze et al., 2012). Empathy can be divided into cognitive and affective components (Cuff et al., 2016). The affective component is called emotional contagion or emotional empathy (Rochat, 2023) and is a precursor of empathy that contributes to relationship quality (Kyranides et al., 2024). Emotional contagion is defined as “the tendency to automatically mimic and synchronize facial expressions, vocalization, postures, and movements with those of another person and, consequently, to converge emotionally” (Hatfield et al., 1992). This emotional contagion is achieved by two processes: (1) inferring or estimating other’s feelings and (2) emotionally resonating with an estimation of other’s feelings. Emotional resonance can be defined as feeling the same way as others. However, as it is sometimes impossible to understand others’ exact feelings, emotional resonance refers to the results of inference.

Previous studies have suggested that many types of mental states and social interactions, including affective and cognitive empathy, change with age (Sze et al., 2012; O'Brien et al., 2013; Khanjani et al., 2015; Gutiérrez-Cobo et al., 2023). For instance, self-reported empathy changes in an inverse-U-shaped pattern across age (O'Brien et al., 2013). That is, adults around 50–60 years of age have greater empathy than young adults and older adults (O'Brien et al., 2013). As social interaction and prosocial behavior are associated with psychological and physical health benefits (Brown and Brown, 2015), understanding how emotional contagion alters through aging and specific experiences would provide important health implications for older adults. However, the degree to which aging affects these states depends on the types of capability, function, or object (Deary et al., 2009; Sze et al., 2012). For instance, cognitive function decreases but emotional empathy and prosocial behavior increase with aging (Deary et al., 2009; Sze et al., 2012). In addition, older adults favor positive and avoid negative information in their attention and memory (Mather and Carstensen, 2005). Therefore, clarifying the effect of aging from various viewpoints is necessary.

Previous studies have investigated social interaction based on child–mother relationships (Schneider et al., 2001; Soltis, 2004). For example, cries from an infant effectively elicit the mother’s attention, proximity, and solicitude (Swain et al., 2007; Rodrigo et al., 2011); thus, behavioral responses are induced, such as distracting, nurturing, or displaying affection to the infant (Zeifman, 2001; Bornstein et al., 2017). That is, emotional contagion involving inference and emotional resonance for infants forms the basis of the motivation to avoid causing harm, as a response to infant cries (Soltis, 2004). Such experience-dependent responses are also important for childcare stress tolerance (Kim, 2016). Lower emotional contagion or resonance in mothers has been associated with a risk of child neglect or maltreatment (de Paúl et al., 2008; Kurth et al., 2011). Additionally, an increased emotional contagion in mothers has been linked to the well-being and development of infants (Boorman et al., 2019). Therefore, these responses may be a strategy of mammalian evolution, ensuring that infants are appropriately cared for and increasing their survival rates (Newman, 1985; Lingle et al., 2012). These changes in mothers are induced by hormones during pregnancy and associated with long-lasting structural plasticity in the brain (Hoekzema et al., 2017). These changes would be associated with appropriate childcare including the processing of baby or infant information (Hoekzema et al., 2017). For example, in first-time mothers, brain response in the auditory cortex was greater when hearing infant crying than adult crying (Hoegholt et al., 2023). However, professional childcare also increases sensitivity to crying (Corvin et al., 2022). Therefore, both childbirth and childcare can change emotional contagion for infants. However, it remains unclear whether the emotional contagion involving inference and emotional resonance for infants changes with age. It is also unclear whether the age-dependent changes in the emotional contagion for infants are influenced by the experiences of childbearing and childrearing.

In Japan, the concept of “the grandchild is the apple of their grandparents’ eye” is common and refers to the extreme fondness that grandparents have for their grandchildren. Interactions with infants induce positive attitudes in older adults (Cohen-Mansfield et al., 2010). The unique effects of interactions with infants observed in older adults may stem from increased emotional contagion for infants. As emotional contagion would be stronger among relatives and familiar others (Gonzalez-Liencres et al., 2014), the positive effect of infant-older adult interaction might be smaller in older adults without childbirth or childcare because of less experience of interaction with children or infants. Therefore, investigating the characteristics of the effect of age and parous-experience on emotional contagion is necessary to maximize the positive effects of the infant-older adult interaction, as it has been suggested that some older adults feel lonely owing to an isolated lifestyle (Pinquart and Sörensen, 2001). That is, it can be predicted whether the effect of interaction with infants on mental states is comparable between parous and nulliparous old women. This evidence would provide useful information, improving older adults’ subjective well-being or quality of life by relieving mental loneliness or isolation.

Previous neuroimaging studies have suggested that positive and negative emotions are processed in different brain regions (Kim and Hamann, 2007). In addition, changes in brain responses in association with aging vary between positive sounds and negative sounds (Mather et al., 2004). A recent previous study has demonstrated that the degree of postpartum depressive symptoms was associated with self-reported negative perception when hearing infant crying but was not associated with self-reported positive perception when hearing infant laughter (Karreman et al., 2023). However, many psychological studies on infant sounds have focused on infant cries. Hence, we used three types of infant sounds, laughter, cooing, and crying, as positive, neutral, and negative infant sounds, respectively. We tested four hypotheses: (1) Emotional resonance for estimated infant valence is increased in old women compared with young women, as previous studies have suggested that feelings for others in older adults alter in an age-dependent manner (O'Brien et al., 2013); (2) An increase of emotional resonance for estimated infant valence in old women is dependent on their parous experience (Bornstein et al., 2017); (3) The effects of aging and parous experience on emotional resonance for estimated infant valence vary depending on types of the infant sounds (Mather et al., 2004; Karreman et al., 2023); and (4) the effects of aging on emotional resonance for estimated infant valence are observed with positive laughing sounds because older adults favor positive and avoid negative information in their attention and memory (Mather and Carstensen, 2005).

## 2 Method

### 2.1 Participants

A total of 252 female volunteers participated in this study. Participants responded to a web-based questionnaire via Google Forms and assessed valence for three types of infant sounds (laughter, cooing, and crying). The participants were recruited via a cloud-sourcing platform. Data from 11 participants were excluded from the analyses because they did not respond appropriately to the catch trials (as described in the Procedure section). Finally, data from 241 participants comprising 60 young nulliparous women (mean age = 31 ± 5 years, range: 22–39 years), 60 young parous women (mean age = 31 ± 5 years, range: 22–39 years), 60 old nulliparous women (mean age = 63 ± 4 years, range: 60–75 years), and 61 old parous women (mean age = 64 ± 4 years, range: 60–77 years) were used for the analyses.

All participants received a detailed explanation of the experimental procedures on the first page of the Google Form. Informed consent for participation was indicated by clicking the start button. This study was conducted in accordance with the Declaration of Helsinki, and the experimental procedures were approved by the Ethics Review Committee for Medical and Health Research involving Human Subjects, Ritsumeikan University (BKC-LSMH-2021-083). We paid 1,000 JPY (about 6.7 USD when 1 USD corresponds to 150 JPY) per participant.

### 2.2 Infant sounds

Infant sound files consisted of three types of infant sounds: laughter, cooing, and crying. The infant sounds were originally collected from 14 acquaintances of the authors who had children between 4 months and 1 year of age. The acquaintances used an iPhone/iPad (Apple Inc.) or a digital voice recorder (ICD-UX560F, SONY) to collect infant sounds during daily activities. We asked the acquaintances to record infant sounds in as quiet an environment as possible. We also asked them to set the voice recorder as close to their infant as possible. Subsequently, the three types of sound were manually extracted from the recorded auditory data. Following this, 15-s sound files were created using Adobe Audition^1^ to control the effect of sound length. For cases where the natural length of laughter, cooing, and crying was shorter than 15 s, files were combined into one 15-s file or repeated using the *remix* function in Adobe Audition which function automatically rearranges any sounds to fit any duration. Additionally, the volume was adjusted, and background noise was reduced using the *hard limiter* and *denoise* functions in Adobe Audition. A total of 150 sound files (i.e., 50 laughter, 50 cooing, and 50 crying) were selected by pre-screening the files, excluding files with unnatural sounds, unclear sounds, or files with annoying noises. In the pre-screening, files with intermixed sounds of different types (e.g., cooing and crying) were also excluded. The authors and research assistants conducted the pre-screening.

### 2.3 Procedure

**Figure 1:**
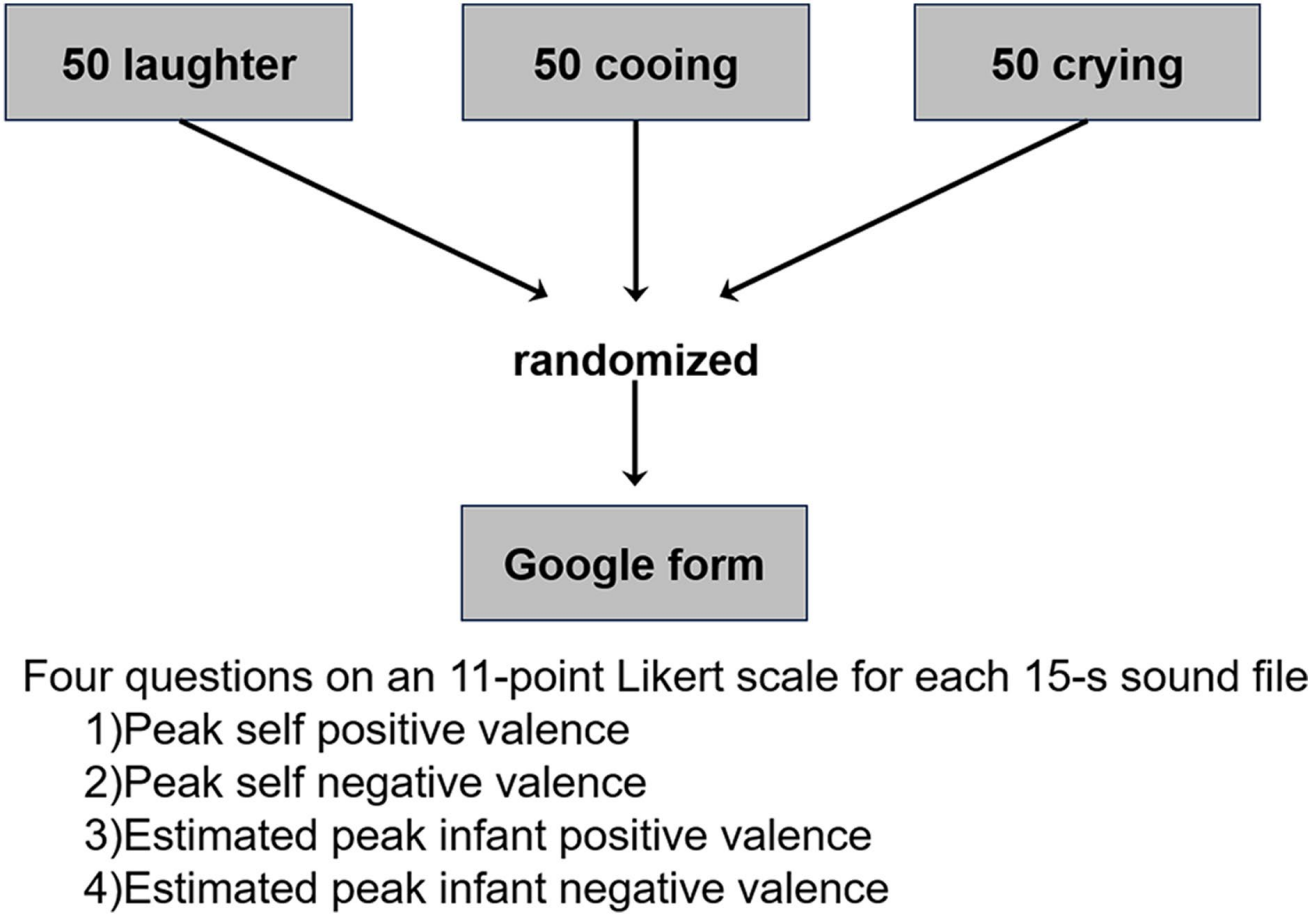
Schematics of experimental design and the list of questions.

The participants responded to four questions on an 11-point Likert scale, for each sound file. The questions were regarding: (1) self positive valence; (2) self negative valence; (3) estimated infant positive valence; and (4) estimated infant negative valence. For positive valence, 0 denotes “extremely negative” and 10 denotes “extremely positive.” Conversely, for negative valence, 0 denotes “extremely positive” and 10 denotes “extremely negative.” We asked the participants to evaluate the peak positive and negative valences because it was difficult to evaluate the mean valence of a 15-s sound file from our pilot study. That is, evaluating the peak positive and negative valences is easier than the mean valence. Note that the infant sounds fluctuated during 15 s (i.e., they were not constant). Therefore, the peak positive and negative valences are not identical. In addition, if we used one valence scale, we were concerned that some participants would be confused about evaluating the mean or peak valance even if we explicitly asked them to evaluate the peak positive/negative valences. The 150 sound files were set in a random order in a Google Form (Figure 1). To improve data quality, four catch trials were set in the 9th, 52nd, 90th, and 141st sound files. Thus, participants completed 154 files in total. In two catch trials, a voice instruction by an adult woman was played as follows: “Please respond to all questions with zero instead of the score for infant sounds.” In the other two catch trials, a voice instruction was played as follows: “Please respond to all questions with ten instead of the score for infant sounds.” Therefore, participants had to respond to all questions with zero or ten in the four catch trials. Participants were excluded if the instructions were not followed at any instance. To reduce a possible order effect, we created four versions of the Google Form with different file orders and assigned one-fourth of each group to each Google Form.

Before responding to the questionnaire for infant sounds, participants responded to several questions regarding: (1) the number of children they had; (2) the age of their children; (3) the gender of their children; (4) the number of grandchildren they had; (5) whether they lived with their children or not; (6) the frequency of meeting their children, and the distance between their homes if they answered “living separately” on question 5; and (7) their preference for infants. The infant preference was indicated on an 11-point Likert scale (0 denotes “extremely dislike” and 10 denotes “extremely like”).

The questionnaire took approximately 60–90 min to complete. Participants were allowed to rest whenever they wished.

### 2.4 Data analysis

To evaluate emotional resonance, Spearman’s correlation coefficients between self and estimated infant valence were calculated for each type of sound. The correlation coefficient may become larger when a woman feels positive after hearing an infant’s sound and simultaneously evaluates the child as feeling positive as well. We adopted correlation analysis rather than the use of differences between self and estimated valence because “zero difference” does not necessarily reflect high emotional resonance. For instance, if a participant always rated “1” for both self and estimated valence in a type of sound (e.g., cooing), the difference becomes zero. However, we do not think that the participant has higher emotional resonance. We used non-parametric Spearman’s correlation analyses because of the discrete data. Correlation coefficients across four groups were then tested using the Kruskal–Wallis test for each type of sound. *Post hoc* analyses were determined using Mann–Whitney *U* tests with the Bonferroni correction.

To compare self valence or inference (i.e., estimated infant valence) among groups, the means of self and estimated infant valences were calculated for each type of sound in each individual, respectively. The individual scores across all four groups were tested using the Kruskal–Wallis test. *Post hoc* analyses utilized Mann–Whitney U tests with the Bonferroni correction. Four comparisons were considered: the factors of experience (i.e., nulliparous vs. parous) and age (i.e., young vs. old). Therefore, we did not test for differences between young parous and old nulliparous women, or between young nulliparous and old parous women in the *post hoc* analyses (e.g., adjusted *p* = 0.05 equal to uncorrected *p* = 0.0125). The effect size was estimated using η^2^ for the Kruskal–Wallis test and using *r* = z/sqrt(N) for the Mann–Whitney *U*-test, where z is the z-statistic estimated by the Matlab function ranksum and N is the number of samples (Fujii et al., 2020).

## 3 Results

The participants’ information is summarized in Table 1. Age was comparable between parous and nulliparous women in the young and old groups (young: *U* = 1757.5, r = 0.020, uncorrected *p* = 0.825; old: *U* = 1705.5, *r* = 0.059, uncorrected *p* = 0.516). The young parous women had 1.65 ± 0.71 (range: 1–4) children (age range: 0–15 years). The old parous women had 2.03 ± 0.91 (range: 1–5) children (age range: 23–50 years) and 1.43 ± 1.87 (range: 0–8) grandchildren. One data regarding the age of the child in the old parous women group was excluded because an old parous woman (62 years old) answered that the child was 1 year old. Two young parous women lived separately from their children, although they did live close to their children (within 4 km) and met every day.

**Table 1:** Participants information.

	Young nulliparous women	Young parous women	Old nulliparous women	Old parous women
Age (years)	31 ± 5	31 ± 5	63 ± 4	64 ± 4
Number of children	NaN	1.65 ± 0.71	NaN	2.03 ± 0.91
Age of their children (years)	NaN	0–15	NaN	23–50
Number of grandchildren	NaN	NaN	NaN	1.43 ± 1.87
Preference for infants	6.43 ± 2.45	8.38 ± 1.40	6.73 ± 2.67	7.70 ± 2.02

### 3.1 Group comparison for infant preference

**Figure 2:**
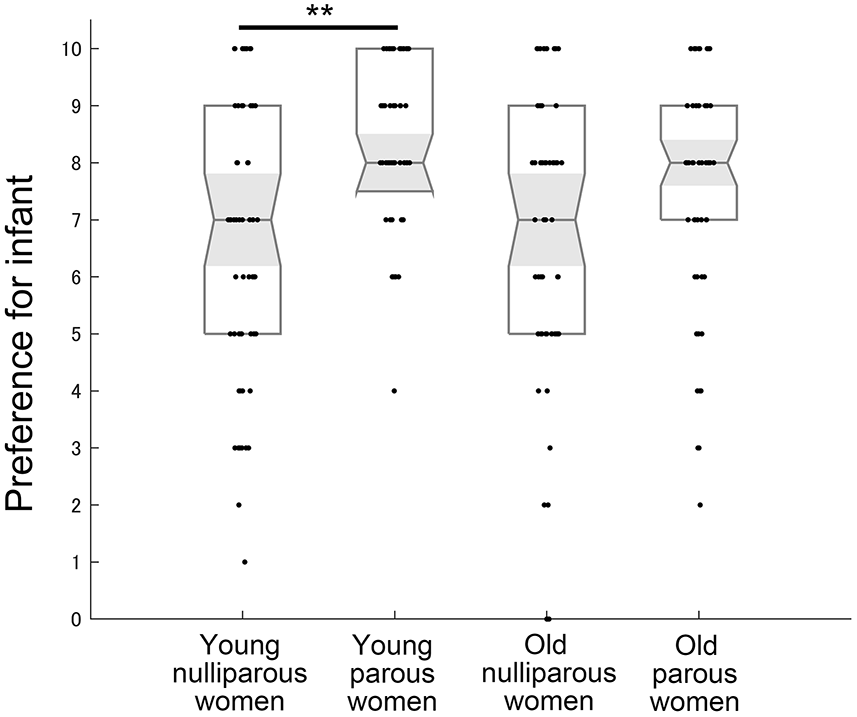
The preference for infants across groups. 10 indicates “extremely like” and 0 indicates “extremely dislike.” Medians, interquartile ranges, 95% confidence intervals of the median, and individual plots are shown. ** indicates *p* < 0.01 with the Bonferroni correction.

The analysis for infant preference using the Kruskal–Wallis test demonstrated a significant difference across groups (χ^2^ = 24.414, η^2^ = 0.102, *p* < 0.001) (Figure 2). The preference for infants in the young group was significantly greater in parous women than in nulliparous women (*U* = 950, r = 0.412, *p* < 0.001). In the old group, the preference for infants did not differ between parous and nulliparous women (*U* = 1,462, *r* = 0.176, *p* = 0.212). In the comparison within parous or nulliparous women, young and old women did not differ significantly (nulliparous young vs. nulliparous old: *U* = 1,632, *r* = 0.081, *p* > 0.999; parous young vs. parous old: *U* = 1,521, *r* = 0.149, *p* = 0.407).

### 3.2 Emotional resonance

#### 3.2.1 Relationship between self and estimated positive valences of laughter

**Figure 3:**
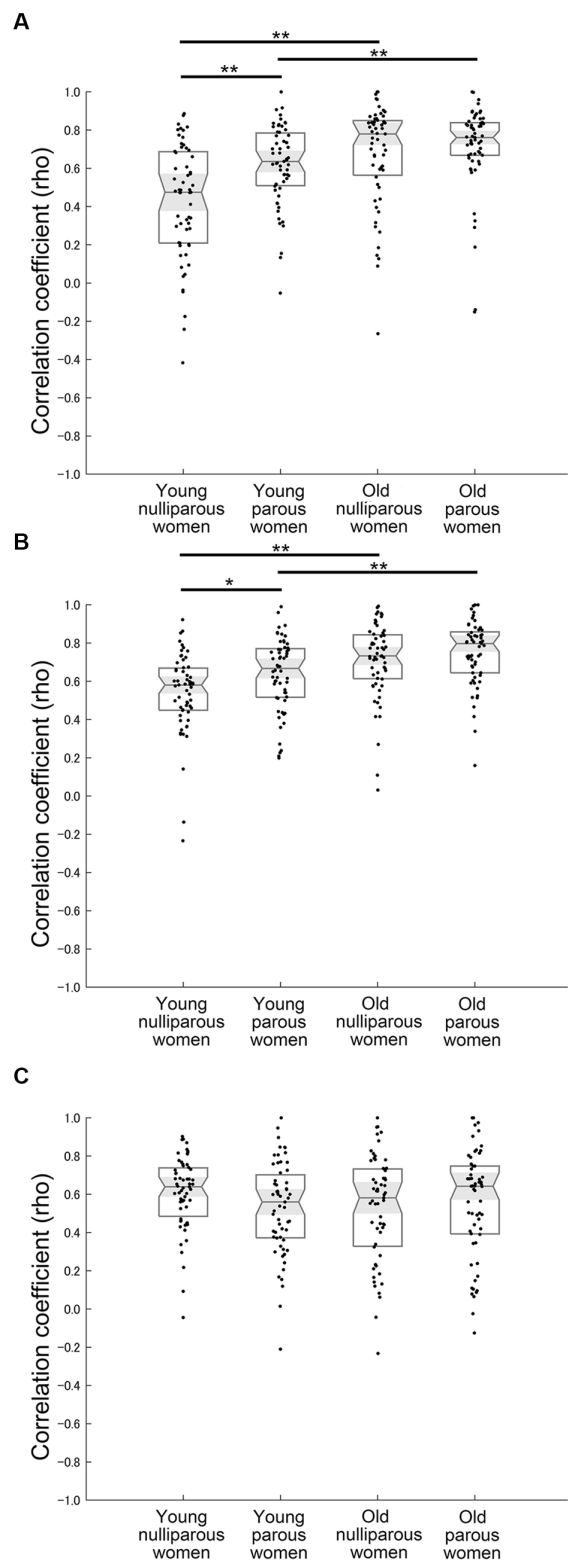
Correlational results. **(A)** Relationship between self and estimated infant positive valence for infant laughter. **(B)** Relationship between self and estimated infant positive valence for infant cooing. **(C)** Relationship between self and estimated infant negative valence for infant crying. * and ** Indicate *p* < 0.05 and *p* < 0.01 with the Bonferroni correction, respectively.

The analysis for correlation coefficients between the self and estimated infant positive valence using the Kruskal–Wallis test demonstrated a significant difference across groups in laughter (χ^2^ = 41.521, η^2^ = 0.177, *p* < 0.001) (Figure 3A and Table 2a). *Post hoc* tests revealed that the correlation coefficients of laughter were greater in young parous women than in young nulliparous women (*U* = 1,001, *r* = 0.334, *p* = 0.001). In addition, the correlation coefficients of laughter in old women were greater than in young women (parous: *U* = 1088.5, *r* = 0.289, *p* = 0.006; nulliparous: *U* = 791, *r* = 0.437, *p* < 0.001), respectively.

**Table 2:** Summary of correlation analyses and comparisons for valences.

			Young nulliparous women (YN)	Young parous women (YP)	Old nulliparous women (ON)	Old parous women (OP)	Significant differences
Relationship between self and estimated infant positive valences	a	Laughter	0.47 (0.21–0.69)	0.64 (0.51–0.78)	0.78 (0.56–0.85)	0.76 (0.67–0.84)	YN < YP; YN < ON; YP < OP
	b	Cooing	0.58 (0.45–0.67)	0.67 (0.52–0.77)	0.73 (0.61–0.84)	0.80 (0.64–0.86)	YN < YP; YN < ON; YP < OP
	c	Crying	0.58 (0.45–0.73)	0.54 (0.34–0.67)	0.57 (0.35–0.74)	0.60 (0.50–0.74)	n.s.
Relationship between self and estimated infant negative valences	d	Laughter	0.39 (0.21–0.59)	0.56 (0.47–0.70)	0.69 (0.50–0.84)	0.72 (0.58–0.83)	YN < YP; YN < ON; YP < OP
	e	Cooing	0.55 (0.41–0.70)	0.65 (0.52–0.73)	0.71 (0.48–0.82)	0.70 (0.58–0.84)	YN < ON; YP < OP
	f	Crying	0.64 (0.49–0.74)	0.56 (0.37–0.70)	0.58 (0.33–0.73)	0.64 (0.39–0.75)	n.s.
Self positive valence	g	Laughter	7.25 (5.82–8.58)	8.52 (7.95–9.48)	7.69 (6.19–8.65)	8.30 (7.11–9.24)	YN < YP
	h	Cooing	5.85 (4.78–7.94)	6.55 (5.96–8.78)	6.36 (5.69–7.52)	7.10 (5.93–8.01)	YN < YP
	i	Crying	3.30 (1.76–4.84)	3.74 (2.95–5.55)	3.80 (2.58–5.00)	3.56 (2.53–4.83)	n.s.
Self negative valence	j	Laughter	2.78 (1.43–5.23)	1.61 (0.48–2.44)	2.45 (1.36–3.96)	1.70 (0.74–3.21)	YN > YP
	k	Cooing	3.92 (2.12–5.48)	3.22 (1.03–4.19)	3.64 (2.44–4.59)	2.68 (1.73–4.18)	YN > YP
	l	Crying	7.64 (5.97–8.70)	6.10 (4.61–7.52)	6.49 (5.14–7.69)	6.48 (4.93–7.74)	YN > YP; YN > ON
Estimated infant positive valence	m	Laughter	8.40 (7.72–9.01)	8.96 (8.25–9.30)	8.18 (7.23–8.85)	8.50 (7.82–9.19)	YN < YP
	n	Cooing	5.63 (5.07–6.37)	5.89 (5.50–6.36)	6.03 (5.66–7.00)	6.44 (5.94–7.33)	YN < ON; YP < OP
	o	Crying	1.89 (1.14–2.69)	2.19 (1.66–2.78)	2.50 (1.58–3.15)	2.10 (1.57–2.62)	n.s.
Estimated infant negative valence	p	Laughter	1.86 (1.04–2.91)	1.42 (0.73–2.14)	2.06 (1.19–2.91)	1.66 (0.94–2.59)	n.s.
	q	Cooing	4.48 (3.89–5.02)	3.99 (3.17–4.86)	3.86 (2.97–4.40)	3.56 (2.53–4.37)	YN > ON
	r	Crying	8.62 (7.86–9.16)	8.12 (7.36–8.79)	7.82 (7.07–8.38)	7.96 (7.63–8.44)	YN > ON
Relationship between infant preference and emotional resonance	s	Laughter	0.50	0.04	0.31	−0.05	
	t	Cooing	0.29	−0.02	0.26	0.06	
	u	Crying	−0.18	−0.16	0.10	−0.20	
Relationship between infant preference and self positive valence	v	Laughter	0.60	0.20	0.67	0.46	
	w	Cooing	0.53	0.14	0.57	0.37	
	x	Crying	0.45	0.23	0.13	0.39	
Relationship between infant preference and self nevative valence	y	Laughter	−0.56	−0.21	−0.67	−0.44	
	z	Cooing	−0.49	−0.18	−0.56	−0.35	
	aa	Crying	−0.54	−0.29	−0.15	−0.39	
Relationship between infant preference and estimated infant positive valence	bb	Laughter	0.27	0.05	0.53	0.30	
	cc	Cooing	0.27	0.16	0.33	0.27	
	dd	Crying	0.05	0.19	−0.10	0.14	
Relationship between infant preference and estimated infant nevative valence	ee	Laughter	−0.20	−0.10	−0.43	−0.23	
	ff	Cooing	−0.19	−0.08	−0.26	−0.17	
	gg	Crying	−0.12	−0.27	0.17	−0.11	

Relationships were calculated using Spearman’s correlation (i.e., rho). For a to *r*, the median, first quartile, and third quartile are shown.

#### 3.2.2 Relationship between self and estimated negative valences of laughter

The analysis for correlation coefficients between the self and estimated infant negative valence using the Kruskal–Wallis test demonstrated a significant difference across groups in laughter (χ^2^ = 37.468, η^2^ = 0.160, *p* < 0.001) (Table 2d). *Post hoc* tests revealed that the correlation coefficients of laughter were greater in young parous women than in young nulliparous women (*U* = 883.5, *r* = 0.258, *p* = 0.004). In addition, the correlation coefficients of laughter in old women were greater than in young women (parous: *U* = 1,000, *r* = 0.264, *p* = 0.015; nulliparous: *U* = 824, *r* = 0.421, *p* < 0.001), respectively.

#### 3.2.3 Relationship between self and estimated positive valences of cooing

The analysis for correlation coefficients between the self and estimated infant positive valence using the Kruskal-Wallis test demonstrated a significant difference across groups in cooing (χ^2^ = 40.486, η^2^ = 0.169, *p* < 0.001) (Figure 3B and Table 2b). *Post hoc* tests revealed that the correlation coefficients of cooing were greater in young parous women than in young nulliparous women (*U* = 1,202, *r* = 0.246, *p* = 0.028). In addition, the correlation coefficients of cooing in old women were greater than in young women (parous: *U* = 1,176, *r* = 0.308, *p* = 0.003; nulliparous: *U* = 816, *r* = 0.434, *p* < 0.001), respectively.

#### 3.2.4 Relationship between self and estimated negative valences of cooing

The analysis for correlation coefficients between the self and estimated infant negative valence using the Kruskal–Wallis test demonstrated a significant difference across groups in cooing (χ^2^ = 23.153, η^2^ = 0.098, *p* < 0.001) (Table 2e). *Post hoc* tests revealed that the correlation coefficients of cooing in old women were greater than in young women (parous: *U* = 1,157, *r* = 0.238, *p* = 0.035; nulliparous: *U* = 1,095, *r* = 0.287, *p* = 0.007), respectively.

#### 3.2.5 Relationship between self and estimated positive valences of crying

The correlation coefficients between the self and estimated infant positive valence of crying did not differ among groups (χ^2^ = 4.012, η^2^ = 0.017, *p* = 0.260) (Table 2c).

#### 3.2.6 Relationship between self and estimated negative valences of crying

The correlation coefficients between the self and estimated infant negative valence of crying did not differ among groups (χ^2^ = 4.072, η^2^ = 0.017, *p* = 0.254) (Figure 3C and Table 2f).

### 3.3 Self valences

#### 3.3.1 Self positive valence of laughter

**Figure 4:**
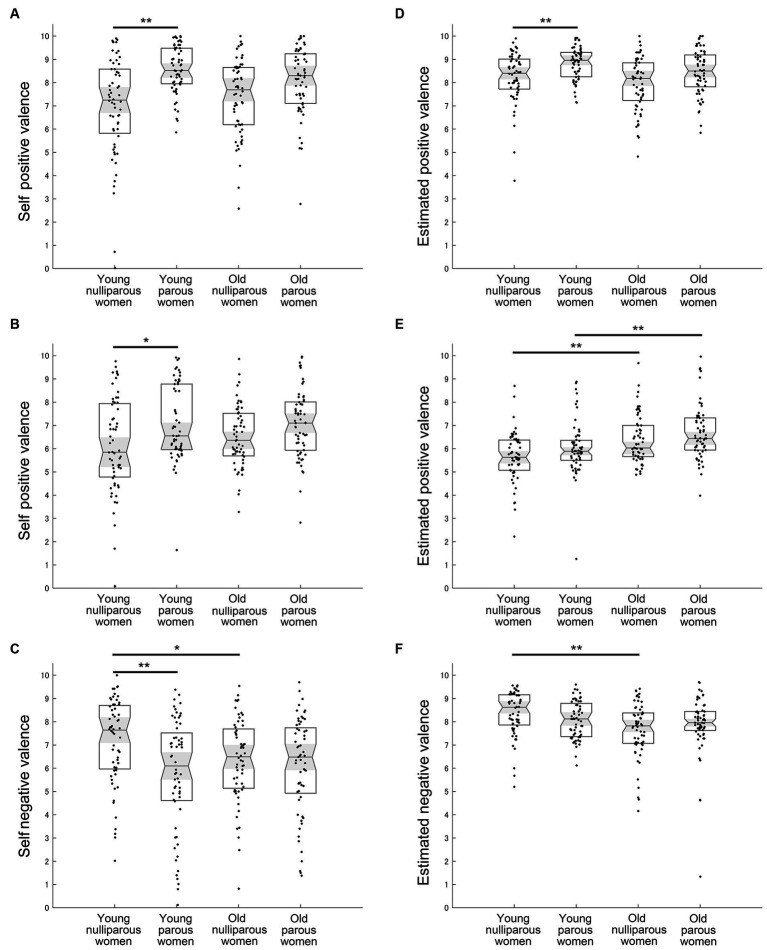
Mean results of self and estimated valance. **(A)** Self positive valence for infant laughter. **(B)** Self positive valence for infant cooing. **(C)** Self negative valence for infant crying. **(D)** Estimated infant positive valence for infant laughter. **(E)** Estimated infant positive valence for infant cooing. **(F)** Estimated infant negative valence for infant crying. Medians, interquartile ranges, 95% confidence intervals of the median, and individual plots are shown. * and ** Indicate *p* < 0.05 and *p* < 0.01 with the Bonferroni correction, respectively.

The analysis for the self positive valence of laughter using the Kruskal-Wallis test demonstrated a significant difference across groups (χ^2^ = 27.578, η^2^ = 0.115, *p* < 0.001) (Figure 4A and Table 2g). The self positive valence for laughter was significantly greater in young parous women than in young nulliparous women (*U* = 948.5, *r* = 0.408, *p* < 0.001). In the old group, self positive valence for laughter tended to be greater in parous women than nulliparous women (*U* = 1360.5, *r* = 0.221, *p* = 0.060). In the comparison within parous and nulliparous women, young and old women did not differ significantly (young parous vs. old parous: *U* = 1,497, *r* = 0.157, *p* = 0.339; young nulliparous vs. old nulliparous: *U* = 1651.5, *r* = 0.071, *p* > 0.999).

#### 3.3.2 Self negative valence of laughter

The analysis for the self negative valence of laughter using the Kruskal-Wallis test demonstrated a significant difference across groups (χ^2^ = 22.029, η^2^ = 0.092, *p* < 0.001) (Table 2j). The self negative valence for laughter was significantly greater in young parous women than in young nulliparous women (*U* = 1015.5, *r* = 0.376, *p* < 0.001).

#### 3.3.3 Self positive valence of cooing

The analysis for the self positive valence of cooing demonstrated a significant difference across groups (χ^2^ = 12.391, η^2^ = 0.052, *p* = 0.006) (Figure 4B and Table 2h). The self positive valence for cooing in the young group was significantly greater in parous women than in nulliparous women (*U* = 1272.5, *r* = 0.253, *p* = 0.023). In the old group, the self positive valence for cooing did not differ between parous and nulliparous women (*U* = 1412.5, *r* = 0.197, *p* = 0.123). Furthermore, in the comparison within parous or nulliparous women, young and old women did not differ significantly (young parous vs. old parous: *U* = 1774, *r* = 0.026, *p* > 0.999; young nulliparous vs. old nulliparous: *U* = 1585.5, *r* = 0.103, *p* > 0.999).

#### 3.3.4 Self negative valence of cooing

The analysis for the self negative valence of cooing using the Kruskal-Wallis test demonstrated a significant difference across groups (χ^2^ = 10.437, η^2^ = 0.043, *p* = 0.015) (Table 2k). The self negative valence for cooing in the young group was significantly smaller in parous women than in nulliparous women (*U* = 1313.5, *r* = 0.233, *p* = 0.043).

#### 3.3.5 Self positive valence of crying

The self positive valence of crying did not differ across groups (χ^2^ = 6.612, η^2^ = 0.027, *p* = 0.085) (Table 2i).

#### 3.3.6 Self negative valence of crying

The analysis for self negative valence of crying demonstrated a significant difference across groups (χ^2^ = 13.335, η^2^ = 0.057, *p* = 0.004) (Figure 4C and Table 2l). The self negative valence for crying in the young group was significantly greater in nulliparous women than in parous women (*U* = 1179.5, *r* = 0.297, *p* = 0.005). In the old group, the self negative valence for crying did not differ between parous women and nulliparous women (*U* = 1756.5, *r* = 0.034, *p* > 0.999). The self negative valence for crying did not differ between young parous women and old parous women (*U* = 1709.5, *r* = 0.057, *p* > 0.999) whereas young nulliparous women felt more negative than old nulliparous women (*U* = 1320.5, *r* = 0.230, *p* = 0.048).

### 3.4 Estimated infant valences

#### 3.4.1 Estimated infant positive valence for laughter

The analysis for the estimated infant positive valence of laughter demonstrated a significant difference across groups (χ^2^ = 21.164, η^2^ = 0.088, *p* < 0.001) (Figure 4D and Table 2m). The estimated infant positive valence for laughter in the young group was significantly greater for parous women than nulliparous women (*U* = 1180.5, *r* = 0.297, *p* = 0.005). In the old group, the estimated infant positive valence for laughter tended to be greater in parous women than nulliparous women (*U* = 1,370, *r* = 0.217, *p* = 0.069). In the comparison within parous or nulliparous women, young and old women did not differ significantly (young parous vs. old parous: *U* = 1452.5, *r* = 0.178, *p* = 0.203; young nulliparous vs. old nulliparous: *U* = 1512.5, *r* = 0.138, *p* = 0.528).

#### 3.4.2 Estimated infant negative valence for laughter

The analysis for the estimated infant positive valence of laughter demonstrated a significant difference across groups (χ^2^ = 10.648, η^2^ = 0.044, *p* = 0.014) (Table 2p). No significant result was found in *Post hoc* tests (*p* > 0.05).

#### 3.4.3 Estimated infant positive valence for cooing

The analysis for the estimated infant positive valence of cooing demonstrated a significant difference across groups (χ^2^ = 27.463, η^2^ = 0.114, *p* < 0.001) (Figure 4E and Table 2n). The estimated infant positive valence for cooing in both age groups did not differ between parous and nulliparous women (young nulliparous vs. young parous: *U* = 1483.5, *r* = 0.151, *p* = 0.389; old nulliparous vs. old parous: *U* = 1,502, *r* = 0.154, *p* = 0.358). In the comparison within parous and nulliparous women, old women scored higher than young women (young parous vs. old parous: *U* = 1,151, *r* = 0.320, *p* = 0.002; young nulliparous vs. old nulliparous: *U* = 1164.5, *r* = 0.304, *p* = 0.003).

#### 3.4.4 Estimated infant negative valence for cooing

The analysis for the estimated infant negative valence of cooing demonstrated a significant difference across groups (χ^2^ = 24.849, η^2^ = 0.104, *p* < 0.001) (Table 2q). The estimated infant negative valence for cooing in the young nulliparous women felt more negative than old nulliparous women (*U* = 1,041, *r* = 0.363, *p* < 0.001). The estimated infant negative valence for cooing in the young nulliparous women tended to feel more negative than young parous women (*U* = 1328.5, *r* = 0.226, *p* = 0.054).

#### 3.4.5 Estimated infant positive valence for crying

The estimated infant positive valence of crying did not differ across groups (χ^2^ = 5.962, η^2^ = 0.025, *p* = 0.114) (Table 2o).

#### 3.4.6 Estimated infant negative valence for crying

The analysis for the estimated infant negative valence of crying demonstrated a significant difference across groups (χ^2^ = 16.049, η^2^ = 0.067, *p* = 0.001) (Figure 4F and Table 2r). The estimated infant negative valence of crying was significantly greater in young nulliparous women than in old nulliparous women (*U* = 1087.5, *r* = 0.341, *p* < 0.001), whereas there was no difference between young and old parous women (*U* = 1780, *r* = 0.023, *p* > 0.999). In the comparison within young and old women, parous and nulliparous women did not differ significantly regarding the estimated infant negative valence of crying (young parous vs. young nulliparous: *U* = 1,371, *r* = 0.205, *p* = 0.098; old parous vs. old nulliparous: *U* = 1552.5, r = 0.131, *p* = 0.604).

### 3.5 Correlations between variables and self or estimated valences

The relationships between infant preference and self or estimated infant valences are shown in Table 2s-gg. The length of childcare (i.e., the age of oldest children) in the young parous women was not correlated with emotional differences or self valences (Table 3). However, the length of childcare was correlated with estimated infant negative valence of cooing (rho = −0.32, *p* = 0.013) (Table 3n). The relationships between participants’ ages and the degree of emotional resonance, self, or estimated valences are shown in Table 4 as a supplemental result.

**Table 3:** Relationship between the length of childcare and emotional resonance or valences.

			Young parous women
Relationship between the age of oldest children and emotional resonance	a	Laughter	rho = −0.05, *p* = 0.688
	b	Cooing	rho = −0.17, *p* = 0.197
	c	Crying	rho = −0.17, *p* = 0.197
Relationship between the age of oldest children and self positive valence	d	Laughter	rho = −0.02, *p* = 0.865
	e	Cooing	rho = 0.10, *p* = 0.436
	f	crying	rho = 0.08, *p* = 0.539
Relationship between the age of oldest children and self negative valence	g	Laughter	rho = 0.03, *p* = 0.791
	h	Cooing	rho = −0.20, *p* = 0.130
	i	Crying	rho = −0.07, *p* = 0.601
Relationship between the age of oldest children and estimated infant positive valence	j	Laughter	rho = 0.003, *p* = 0.979
	k	Cooing	rho = 0.22, *p* = 0.095
	l	Crying	rho = 0.01, *p* = 0.929
Relationship between the age of oldest children and estimated infant negative valence	m	Laughter	rho = 0.04, *p* = 0.756
	n	Cooing	rho = −0.32, p = 0.013
	o	Crying	rho = −0.08, *p* = 0.519

Uncorrected *p*-values are shown.

**Table 4:** Relationship between participants’ age and emotional resonance or valences.

		Young nulliparous women	Young parous women	Old nulliparous women	Old parous women
Relationship between the participants’ age and emotional resonance	Laughter	rho = 0.283, *p* = 0.119	rho = 0.052, *p* > 0.999	rho = −0.019, *p* > 0.999	rho = 0.362, *p* = 0.020
	Cooing	rho = 0.144, *p* > 0.999	rho = −0.164, *p* = 0.842	rho = 0.061, *p* > 0.999	rho = 0.089, *p* > 0.999
	Crying	rho = −0.013, *p* > 0.999	rho = −0.251, *p* = 0.220	rho = 0.032, *p* > 0.999	rho = 0.144, *p* > 0.999
Relationship between the participants’ age and self valence	Laughter	rho = 0.089, *p* > 0.999	rho = −0.185, *p* = 0.627	rho = −0.082, *p* > 0.999	rho = 0.154, *p* = 0.940
	Cooing	rho = 0.049, *p* > 0.999	rho = 0.017, *p* > 0.999	rho = −0.108, *p* > 0.999	rho = 0.090, *p* > 0.999
	Crying	rho = −0.055, *p* > 0.999	rho = −0.103, *p* > 0.999	rho = −0.019, *p* > 0.999	rho = −0.045, *p* > 0.999
Relationship between the participants’ age and estimated infant valence	Laughter	rho = −0.051, *p* > 0.999	rho = −0.183, *p* = 0.651	rho = −0.141, *p* > 0.999	rho = −0.049, *p* > 0.999
	Cooing	rho = 0.076, *p* > 0.999	rho = 0.022, *p* > 0.999	rho = −0.086, *p* > 0.999	0.138, *p* > 0.999
	Crying	rho = −0.179, *p* = 0.688	rho = −0.025, *p* > 0.999	rho = −0.152, *p* = 0.991	−0.195, *p* = 0.530

Adjusted *p*-values are shown (e.g., adjusted *p* = 0.05 equal to uncorrected *p* = 0.0125).

## 4 Discussion

The emotional resonance for infant laughter and cooing, assessed using correlation coefficients between self and estimated infant valences, were greater in parous women and both groups of old women as compared with those in young nulliparous women. The trends were the same when analyzing negative valences. Thus, emotional resonance for positive infant sounds likely increases with age and experience of childbirth/childcare. The degrees of correlation coefficients for laughter and cooing between old parous and nulliparous women did not differ. Therefore, age-dependent changes in emotional resonance for infant sounds may not be enhanced by parous experience. Interestingly, the degrees of correlation coefficients in both old nulliparous and old parous women were greater than those in young parous women, suggesting that the effect of age on increasing emotional resonance for positive infant sounds is likely stronger than that of parous experience.

Conversely, the correlation coefficient between self and estimated infant valence for crying was comparable among all four groups. Therefore, for the negative infant sounds, emotional resonance for infants would not be changed by aging or parous experience. The unchanged emotional resonance for negative infant crying sounds may be important for sustaining the mental health of young parous women (i.e., mothers). That is, if emotional resonance for crying increases excessively, negative mental states and mental illness would be enhanced by increasing sensitivity for negative crying sounds. A previous study has demonstrated that mental stress and the brain response for infant crying are linked (Kim et al., 2020). As lower emotional contagion or resonance in mothers poses a risk of child neglect or maltreatment (de Paúl et al., 2008; Kurth et al., 2011), it is adverse to have too much or too little emotional resonance for negative infant sounds. Previous studies investigating emotional regulation, which is defined as the maintenance of positive affect and decrease of negative affect, suggest that older adults pay more attention to positive than negative information (Mather and Carstensen, 2003), and thus, healthy older adults can remember positive events more than negative events (Charles et al., 2003). Such positive bias observed in older adults is likely beneficial to improve mental health and well-being (Mather and Carstensen, 2005). Therefore, positive sounds leading to specific changes in emotional resonance for infant sounds might serve a function in improving mental health and well-being in older women. Furthermore, the null result of emotional resonance for crying sounds excludes the possibility that the increased emotional resonance observed in older women merely reflects deteriorated cognitive function, as participants with lower cognitive function might tend to respond with the same scores for self and estimated infant valences.

When we analyzed the mean values of self or estimated infant valence independently, trends in age and parous-experience dependent changes differed among the types of infant sounds and valences. When hearing infant laughter and cooing, young parous women felt more positive than young nulliparous women. These results suggested that experience of childbirth and/or childcare increase positive feelings regarding positive or neutral infant sounds. These behavioral changes in young mothers might be associated with changes in brain structure during pregnancy (Hoekzema et al., 2017). This trend was also observed in old women in this study, although the difference did not reach statistical significance. However, the combined effect of aging and parous experience on self positive valence was not observed for laughter and cooing. Therefore, positive bias for positive infant sounds would be saturated by aging or experiencing childbirth and/or childcare. Notably, the pattern of group differences in the estimated infant positive valence of laughter was different from that of cooing. Therefore, inference (i.e., estimated infant positive valence) for laughter changed in an experience-dependent manner, whereas inference for cooing changed in an age-dependent manner. Estimating an infant’s emotion from hearing cooing might be difficult compared to estimating emotion from hearing laughter. Therefore, as cognitive function decreases with age (Deary et al., 2009), the age-dependent effect of inference would be greater for uncertain infant sounds (i.e., cooing) and biased to be positive rather than negative. In addition, age-related hearing loss might be a potential confounder. Notably, cooing sounds had a relatively lower intonation than laughter. Previous studies suggest that inference is affected by experience of childbirth and/or childcare (Bouchet et al., 2020; Corvin et al., 2022). Thus, the found differences in the estimated infant valence for laughter between young parous and young nulliparous women support previous studies’ results.

When hearing infant negative crying sounds, the self negative valence in young nulliparous women was greater than that in young parous and old nulliparous women. Therefore, after hearing the infant crying, self negative valence decreases in a parous-experience and age-dependent manner. This evidence supports previous studies suggesting that emotional regulation improves with age (Mather and Carstensen, 2005). The estimated infant valence for crying was smaller in old nulliparous women than in young nulliparous women whereas correlation coefficients between self and estimated infant valences were not changed by aging. Therefore, the changes in emotional resonance (assessed by the relationship between self and estimated infant valence) and inference (assessed by estimated infant valence) observed in old women might occur independently. Our findings support previous studies suggesting that these two functions are processed in different neural networks (Zaki and Ochsner, 2012).

In summary, Hypotheses 1, 3, and 4 were confirmed. However, Hypothesis 2, outlining that an increase of emotional resonance for estimated infant valence in old women is dependent on their parous experience, was rejected.

According to the literature, the experience of childcare influences sensitivity to crying (Corvin et al., 2022). Thus, the length of childcare periods could affect the participants’ emotional resonance, or self and estimated infant valences. Supplemental analyses demonstrate that the number of years of childcare was not associated with emotional resonance, or self and estimated infant valences. Therefore, the effect of long-term childcare experience on emotional resonance or valence for infant sounds might be minimal. In addition, the ages of the young women group (i.e., 20–40 years old) did not correlate with the degree of emotional resonance, self, or estimated valences. Therefore, age-dependent changes, which were observed in this study, may only occur after 40 years of age. Future studies should investigate women between 40 and 60 years of age and analyze the inflection point.

This study has some limitations. One limitation is that we cannot claim causality. In this study, sampling bias may have influenced the results of comparison between nulliparous and parous women. That is, it is possible that women who dislike infants and have a negative impression of their sounds do not want to have a child. Infant preference in young nulliparous women was significantly lower than that in young parous women. Therefore, caution is warranted when interpreting the results comparing nulliparous and parous women. However, preference for infants in old nulliparous women was comparable with that in young nulliparous women. Thus, the increased emotional resonance in old nulliparous women cannot be explained by a preference for infants. In addition, we cannot determine whether increased emotional resonance in older women was induced by biological changes, environmental factors, or both. Previous studies have demonstrated that mental states are influenced by many environmental factors such as fitness level, income, and social stress (Allen et al., 2014). In addition, participants who have a history of post-partum depression might have been included. A previous study has suggested that severe stress in a post-partum period influences brain activity and maternal sensitivity during an infant-mother interaction (Kim et al., 2020). Furthermore, we did not assess the length of childcare for other’s children in nulliparous women, although previous studies suggest that recognition of infant for infant cry can be learned through the caregiving experience (Bouchet et al., 2020; Corvin et al., 2022). That is, some nulliparous women might have a professional caregiving experience. Therefore, it is difficult to determine whether the difference between nulliparous and parous women was caused by childcare or childbirth. Hence, future studies using longitudinal designs or randomized controlled trials that collect detailed personal data are needed for clarification. Furthermore, the interpretation of inference should be cautiously considered. In the present study, the ground truth of infant valence is unclear. That is, it is impossible to assess the infant’s actual feelings. Therefore, we were unable to evaluate the accuracy of estimated infant valence. Even if that were the case, the emotional resonance among groups was not influenced by the accuracy of inference because emotional resonance refers to an estimation of other’s feelings. In future studies, the use of classified infant sounds (i.e., an infant is crying because of hunger) is needed to evaluate the capability of inference for infant sounds (Lockhart-Bouron et al., 2023). In addition, the infant age was not completely balanced among the three types of sounds. Investigating the effect of infant age would also be valuable as a previous study has demonstrated that infant cries change with age (Lockhart-Bouron et al., 2023). Furthermore, in the pre-screening, the selected 50 sounds in each type of sound could not be balanced for the number of different infants. For instance, we could not collect high-quality cooing from some infants (e.g., from an infant, 5 laugher, 1 cooing, and 4 crying were selected in the pre-screening). As this heterogeneous distribution of individuals among the three types of sounds may influence the results, caution is warranted when interpreting the results comparing sound types.

In the present study, we only collected data from women; however, previous studies have reported sex and gender differences in empathy (Rochat, 2023). Consequently, investigating old men and young fathers using the same procedure, and comparing results between female and male participants is necessary. In addition, all participants in this study were East Asian (i.e., Japanese). It would be interesting to investigate whether our finding is common in different cultural populations as the response to children crying seems to be common among different cultures (Bornstein et al., 2017).

## 5 Conclusion

This study investigated whether emotional contagion for infant sounds such as laughter, cooing and crying changes with age and in a parous-experience dependent manner. The results indicate that emotional resonance, assessed by correlation coefficients between self and estimated infant valence, increased with age and in a parous-experience dependent manner regarding positive infant sounds (i.e., laughter and cooing), but not regarding negative crying sounds. These findings provide useful knowledge regarding the improvement of well-being for older women by relieving mental loneliness or isolation. That is, the interaction with infants or infant robots could be effective for the well-being and mental states of not only parous but also nulliparous old women.

## Data availability statement

The raw data supporting the conclusions of this article will be made available by the authors, without undue reservation.

## Ethics statement

The studies involving humans were approved by the Ethics Review Committee for Medical and Health Research involving Human Subjects, Ritsumeikan University. The studies were conducted in accordance with the local legislation and institutional requirements. Written informed consent for participation was not required from the participants or the participants' legal guardians/next of kin in accordance with the local legislation and institutional requirements.

## Author contributions

NM: Conceptualization, Data curation, Formal analysis, Investigation, Methodology, Visualization, Writing – original draft, Writing – review & editing. KK: Conceptualization, Formal analysis, Methodology, Supervision, Validation, Writing – review & editing. SS: Conceptualization, Methodology, Validation, Writing – review & editing. TY: Conceptualization, Methodology, Writing – review & editing. YG: Data curation, Investigation, Writing – review & editing. KT: Data curation, Investigation, Writing – review & editing. TI: Project administration, Resources, Supervision, Writing – review & editing.

## Footnotes

^1^ https://www.adobe.com/
